# Chemical induction of DNA demethylation by 5-Azacytidine enhances tomato fruit defense against gray mold through dicer-like protein *DCL2c*

**DOI:** 10.1093/hr/uhae164

**Published:** 2024-06-19

**Authors:** Xiaorong Chang, Liyao Liu, Ziwei Liu, Liping Qiao, Ruixi Shi, Laifeng Lu

**Affiliations:** Tianjin Key Laboratory of Food Quality and Health, State Key Laboratory of Food Nutrition and Safety, College of Food Engineering and Biotechnology, Tianjin University of Science and Technology, Tianjin 300457, China; Tianjin Key Laboratory of Food Quality and Health, State Key Laboratory of Food Nutrition and Safety, College of Food Engineering and Biotechnology, Tianjin University of Science and Technology, Tianjin 300457, China; Tianjin Key Laboratory of Food Quality and Health, State Key Laboratory of Food Nutrition and Safety, College of Food Engineering and Biotechnology, Tianjin University of Science and Technology, Tianjin 300457, China; Tianjin Key Laboratory of Food Quality and Health, State Key Laboratory of Food Nutrition and Safety, College of Food Engineering and Biotechnology, Tianjin University of Science and Technology, Tianjin 300457, China; Tianjin Key Laboratory of Food Quality and Health, State Key Laboratory of Food Nutrition and Safety, College of Food Engineering and Biotechnology, Tianjin University of Science and Technology, Tianjin 300457, China; Tianjin Key Laboratory of Food Quality and Health, State Key Laboratory of Food Nutrition and Safety, College of Food Engineering and Biotechnology, Tianjin University of Science and Technology, Tianjin 300457, China

## Abstract

Postharvest decay, primarily caused by pathogenic fungi in ripening fruits and fresh vegetables, poses a challenge to agricultural sustainability and results in significant economic losses. The regulation of the fruit ripening by DNA methylation has been well demonstrated, while defense response of fruit underlying epigenetic regulation against postharvest decay remains uncertain. In the present study, treatment of tomato fruits with the DNA methyltransferase inhibitor 5-Azacytidine (5-Aza) notably decreased their susceptibility to gray mold. Following 5-Aza treatment, we observed a substantial increase in activities of chitinase (CHI) and glucanase (GLU) in tomato fruits, as well as an increase in the expression of the dicer-like *SlDCL2* gene family. Suppression of *SlDCL2c* through double-stranded RNA-induced RNA interference (RNAi) resulted in a decrease in the expression of chitinases *CHI3, CHI9, Class V chitinase*, and *endochitinase 4* by 71%, 29%, 55%, 64%, as well as glucanases *Cel1, Cel2*, and *GluB* by 19%, 93%, and 87%, respectively. This was accompanied by decreased activities of resistance-related enzymes, including CHI and GLU. The expression levels of genes phenylalanine ammonia-lyase *PAL2*, peroxidase *POD 12*, *POD P7*, *CCR1*, *CYP84A2*, and *COMT* in phenylpropanoid biosynthesis pathway also decreased by 33%, 53%, 18%, 50%, 30%, and 24% in *SlDCL2c-*RNAi fruit, resulting in decreased activities of PAL and POD. Consequently, the lesion diameter of gray mold in *SlDCL2c*-RNAi fruit increased by 55% compared to the control group. Overall, the present study indicated that DNA methyltransferase inhibitor 5-Aza reduces susceptibility of tomato fruit to gray mold through regulation of *DCL2c*-mediated inducible defense response.

## Introduction

Postharvest decay of fruits and vegetables is a significant issue caused by various plant pathogenic microorganisms like fungi and bacteria, leading to substantial food loss during storage, transportation and within the supply chain [1, 2]. The risks associated with pathogenic fungi are a major contributing factor to fruit and food insecurity [3]. Fighting against postharvest decay in fruits and vegetables has been a persistent challenge. Despite advancements in fungicides and modern storage technologies that have significantly prolonged the shelf life of harvested fruit, postharvest losses still range from 5% to over 20% in the United States, and can soar as high as 50% in developing countries [4–6]. However, the application of fungicides has faced obstacles due to pathogen resistance, limited replacement options, and public concerns about the health and environmental impacts of pesticides. Addressing these challenges through innovative and sustainable postharvest management strategies is crucial to ensure food security and minimize greenhouse gas emissions.

Host defense against pathogenic microorganisms is orchestrated by early innate immune responses followed by the activation of adaptive immunity, each providing broad-spectrum and highly specific antimicrobial activity, respectively [7]. Enhancement of the immune responses in infected plants can be achieved through epigenetic changes, such as the activation of the demethylase ROS1 or the loss of RNA-directed DNA methylation (RdDM), which are associated with widespread loss of DNA methylation (hypomethylation) at regions rich in transposable elements [8, 9]. When these enduring effects do not immediately enhance immunity, they may be recognized as ‘priming’: the defense and stress tolerance mechanism is poised to be activated more rapidly and robustly than in the unprimed state when challenged by pathogens, insects, or abiotic stressors [10]. The utilization of dominant resistance genes or transgenes for plant and crop protection is notably simpler than the multifaceted mechanisms in wild species, where multiple defense strategies are generally engaged against various pathogens. To overcome the constraints of the traditional dominant-gene approach, drawing insights from epigenetically controlled immune system networks could lead to the development of more sophisticated and long-lasting disease control strategies in fruits and vegetables [9].

Dicer is a double-stranded RNA (dsRNA)-specific endoribonuclease that plays a vital role in initiating transcriptional and post-transcriptional gene silencing in eukaryotes [7]. It cleaves dsRNAs or single-stranded RNAs with stem-loop structures, such as precursor microRNA, into small RNAs that are 21 to 24 nucleotides in length. Plants have evolved to employ at least four Dicer-like (DCL) proteins, namely DCL1–4, thus achieving diverse functions [11]. Each DCL protein participates in a specific gene silencing pathway, exhibiting some redundancy. DCL1 is the sole Dicer protein responsible for the production of the majority of 21-nt miRNAs, coordinating the processing of pri-miRNA to pre-miRNA and mature miRNA [12]. DCL3 predominantly produces 24-nt repeat-associated siRNAs originating from transposons and repetitive DNA elements, contributing to transcriptional gene silencing (TGS) via RNA-dependent DNA methylation to inhibit the proliferation of these elements [11]. DCL4 serves as the primary generator of 21-nt antiviral siRNAs, as well as endogenous siRNAs like trans-acting siRNAs (tasiRNAs) and phased siRNAs (phasiRNAs) [13]. DCL2 can compensate for the absence of DCL4 and plays a vital role in secondary siRNA-mediated transitive silencing, while it not only serves as a backup to DCL4 but also functions downstream of DCL4 to enhance the RNAi silencing signal [14].

DNA demethylation are critical, but not limited, to host defense; in fact, fruit use such mechanisms for the regulation of ripening. In both dry and ethylene-independent fleshy fruits, the epigenome has played a conserved role in constraining the expression of ripening genes and their orthologues [15, 16]. The repression or loss of function of the DEMETER-like DNA demethylase DML2 has been demonstrated to lead to DNA hypermethylation and the inhibition of ripening in tomato [17, 18]. Yet, despite it being known that the regulation of the fruit ripening is by DNA demethylation, the defense response of fruit underlying epigenetic regulation against postharvest decay remains uncertain. In the present study, we employed the DNA methylation inhibitor 5-Azacytidine (5-Aza) to investigate the effects of DNA demethylation on fruit ripening, defense mechanisms, and the resistance to gray mold disease in tomato fruits. We examined the responses of four *DCL2* genes under conditions of chemically induced DNA hypomethylation and observed a significant upregulation of *SlDCL2c* following 5-Aza treatment. To further elucidate the role of *SlDCL2c* in DNA demethylation-enhanced immunity, we assessed the gene expression and activities of pathogenesis-related (PR) proteins and enzymes in the phenylpropanoid biosynthesis pathway, and the resistance to gray mold disease upon the suppression of *SlDCL2c* via spray-induced gene silencing (SIGS) with dsRNA in tomato fruits.

## Results

### The impact of 5-Aza treatment on gray mold disease resistance in tomato fruits

**Figure 1:**
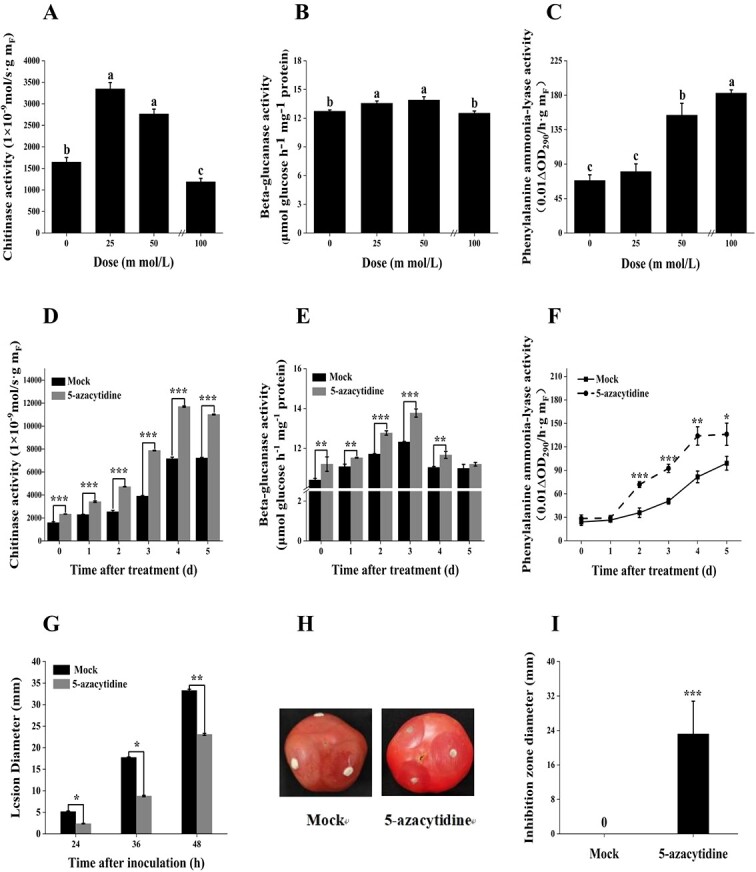
Coating with 5-Azacytidine (5-Aza) enhanced the activity of resistance-related enzymes and gray mold resistance in tomato fruits. The effects of of 5-Aza treatment on the activities of chitinase (**A**), beta-glucanase (**B**), and phenylalanine ammonia-lyase (**C**) in tomato fruits were analysed at various concentrations (0, 25, 50, 100 mmol/L). The impact of 50 mmol/L 5-Aza treatment on the activities of chitinase (**D**), beta-glucanase (**E**), and phenylalanine ammonia-lyase (**F**) in tomato fruits was investigated at different time points. The lesion diameters (**G**) and disease incidence (**H**) of tomato fruits were evaluated against *Botrytis cinerea*. The tomato fruits were coated with 50 mmol/L 5-Aza on their surface for 5 days, while the mock group was treated with distilled water. The antifungal activity of 5-Aza was evaluated against the fungal pathogen using the agar diffusion plate method (**I**). Duncan's multiple comparison test was utilized to compare the mean values among treatments, with differences indicated by different letters (α = 0.05). Student's *t*-test was employed for comparisons between the two treatments, denoted by asterisks at *P* ≤ 0.05 (^*^), *P* ≤ 0.01 (^**^), or *P* ≤ 0.001 (^***^).

PR proteins are a divergent set of antimicrobial molecules that are induced by phytopathogens and defense-related signaling molecules, enhancing the immunity in plants against a wide range of pathogens [19]. To explore the influence of DNA demethylation on the inducible defense response of fruit, the activities of glucanase (GLU, PR2), chitinase (CHI, PR3), as well as phenylalanine ammonia-lyase (PAL), the key enzyme in phenylpropanoid biosynthesis pathway, were monitored in tomatoes treated with the DNA methyltransferase inhibitor 5-Aza. The enhancement of these two PR proteins and PAL activities by 5-Aza was observed to exhibit a concentration-dependent manner

(Fig. 1). As the concentration of 5-Aza increased, a pattern of initially increasing and then decreasing activities was observed for CHI and GLU enzymes in tomato fruits, peaking after treatment with 25 mmol/L and 50 mmol/L of 5-Aza, respectively (Fig. 1A and B). Similarly, the PAL enzyme activity rose with higher concentrations of 5-Aza treatment, reaching their peaks after treatment with 100 mmol/L of 5-Aza (Fig. 1C). In terms of time effect, the activities of CHI and GLU in tomato fruits followed a similar pattern of initial increase followed by decrease, reaching maximum on the 4 d and 5 d of treatment, respectively (Fig. 1D and E). The PAL activity exhibited a consistent increase, peaking on the 5 d after treatment (Fig. 1F). These results indicated 5-Aza notably enhanced the activities of defense enzymes CHI, GLU, and PAL in tomato fruits.

To verify the influence of DNA methylation on susceptibility of postharvest fruit to pathogenic fungi, tomato fruits coated with 5-Aza on their surface and stored for 5 days were challenged with *Botrytis cinerea*, which caused gray mold. The results revealed that the tomato fruits coated with 50 mmol/L of 5-Aza exhibited lesion diameters of 8.78 mm, and 23.09 mm after 36 h, and 48 h of challenge, respectively. In comparison, the lesion diameters of the control group, treated with distilled water, were 17.74 mm and 33.28 mm at the same time intervals, respectively (Fig.  1G and 1H). A significant inhibition of gray mold development in tomato fruits was observed throughout the storage period, with inhibitions of 50.5%, and 30.6% achieved through the application of the 5-Aza coating. Meanwhile, the antifungal activity of 5-Azacytidine was evaluated against the fungal pathogen using the agar diffusion plate method. 5-Aza was found to have a direct inhibitory effect on *B. cinerea.* The diameter of the inhibition zone of 5-Aza was 23.2 ± 7.6 mm against *B. cinerea*, compared to 0 mm in the control groups (Fig. 1I). Therefore, treatment with 5-Aza was supposed to enhance the accumulation of disease resistant genes, directly inhibit the growth of the fungal pathogen, and subsequently reduce the susceptibility of tomato fruit to pathogenic fungi *B. cinerea*.

### Treatment with 5-Aza positively influences the quality of tomato fruits

**Figure 2:**
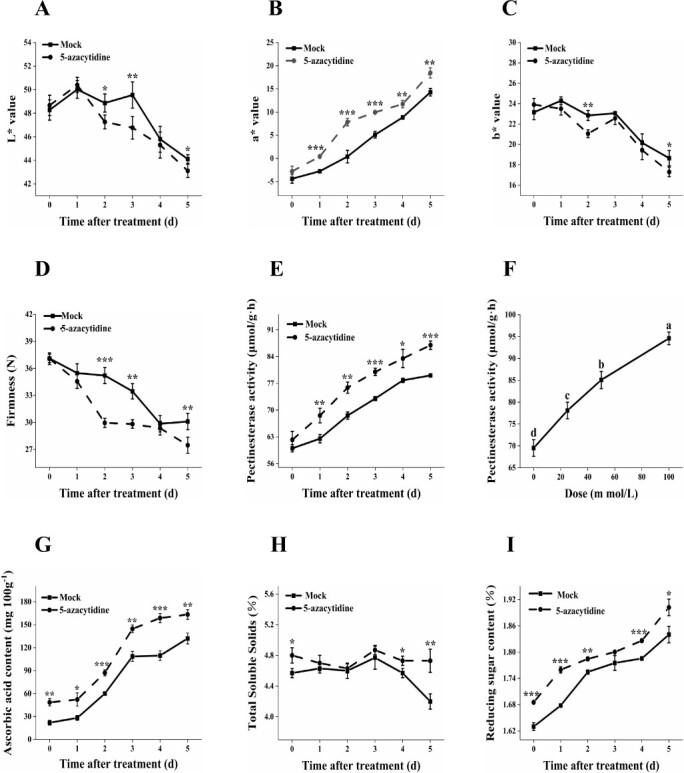
Coating with 5-Azacytidine (5-Aza) improved the quality of tomato fruits. The impact of 5-Aza solution on the fruit color values (**A**, **B**, **C**) and firmness (**D**) of tomato fruits was assessed at various time points. The effects of dose of 5-Aza solution (**E**) (0, 25, 50, 100 mmol/L) and the impact of 50 mmol/L 5-Aza treatment at different time points (**F**) on pectinesterase activity in tomato fruits. Changes in ascorbic acid content (**G**), total soluble solids (**H**), and reducing sugar content (**I**) in tomato fruits following treatment with 50 mmol/L 5-Aza for different durations. Tomato fruits in the 5-Aza treated groups received two applications of 5-Aza, with a two-day interval between each application, while the tomato fruits in the mock group were treated with distilled water.

During the ripening process, fruits undergo a visually striking color transformation from green to yellow to red. This change is primarily driven by the biosynthesis of pigments such as chlorophyll, carotenoids, and anthocyanins, which are intricately regulated by hormonal, genetic, and environmental factors [20]. L^*^ is the chromaticity value that represent lightness (black to white), and a^*^ represents redness to greenness of an object. Here, the L^*^ value and b^*^ value of tomato fruits significantly decreased, while the a^*^ value increased after surface coating with 5-Aza solution compared to the control group (Fig. 2A–C). These changes suggested that the application of 5-Aza drives the color transformation through genetic regulation, promoting the ripening process of tomato fruit, leading to a color change from green to red. The finding was consistent with the report of Zhong *et al.* that exposing tomatoes to the methyltransferase inhibitor 5-Azacytidine led to premature ripening [21]. The mRNA of hallmark ripening gene *psy1*, which encodes phytoene synthase 1, the rate-limiting enzyme in fruit carotenoid synthesis, was detected in the early ripening sector [21]. Moreover, a substantial reduction in fruit hardness was observed over the storage period and with 5-Aza treatment (Fig. 2D). Our investigation revealed a significant increase in the activity of the key fruit softening enzyme, pectinesterase (PE), upon treatment with 5-Aza (Fig. 2E). The augmentation of PE enzyme activity by 5-Aza followed a concentration-dependent pattern, reaching its peak after surface coating with 100 mmol/L of 5-Aza solution (Fig. 2F).

Ascorbic acid (AsA) is a crucial nutrient that affects the primary flavor of tomato fruits and human health. Here, the levels of AsA increased proportionally with treatment concentration, reaching their highest levels at 100 mmol/L 5-Aza treatment (Table S1, see online supplementary material). The AsA content showed an increasing trend, reaching their maximum levels on the 5 d after treatment (Fig. 2G). Similarly, treatment with 5-Azacytidine was observed to significantly decrease the DNA methylation levels of the biosynthetic AsA gene *SlGalUR5* in the fruit, leading to increased accumulation of AsA in both tomato leaves and fruit [22]. Meanwhile, the soluble solid content was observed initially increasing and then decreasing with increasing 5-Aza concentration, peaking at 25 mmol/L 5-Aza treatment (Table S1, see online supplementary material). The levels of reducing sugar increased proportionally with treatment concentration, reaching their highest levels at 100 mmol/L 5-Aza treatment (Table S1, see online supplementary material). Over time, the soluble solid content initially increased before decreasing, reaching its peak on the 4 d of treatment (Fig. 2H). The reducing sugar content showed an increasing trend, reaching their maximum levels on the 5 d of treatment (Fig. 2I). Our findings collectively confirm that after surface coating with 5-Aza, the soluble solid content, ascorbic acid, and reducing sugar content of tomato fruits increased significantly as the fruits ripened.

### Spray-induced DCL2 gene silencing in tomato fruit

**Figure 3:**
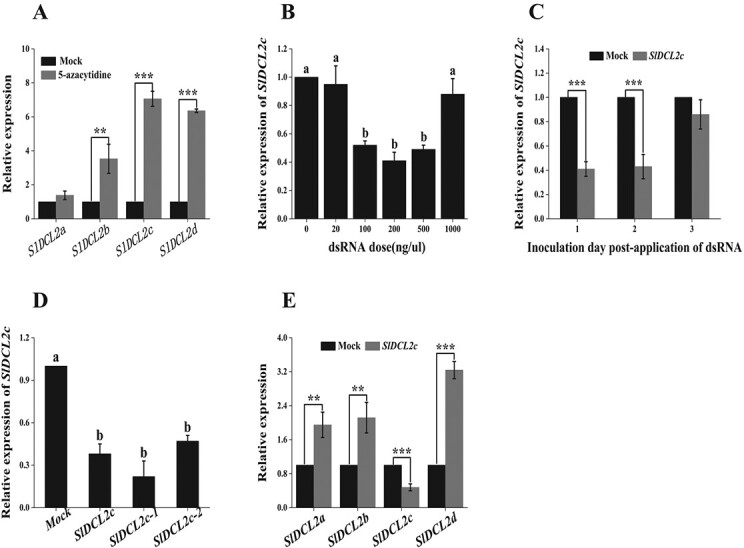
The suppression of *SlDCL2* via spray-induced gene silencing (SIGS) with dsRNA in tomato fruits. (**A**) The expression levels of four genes, *SlDCL2a*, *SlDCL2b*, *SlDCL2c*, and *SlDCL2d* in tomato fruits treated with 5-Aza. The tomato fruits were coated with 50 mmol/L 5-Aza on their surface for 5 days, while the mock group was treated with distilled water. (**B**) Relative expression of the *SlDCL2c* gene in tomato fruits was evaluated after inoculation with different concentrations of SlDCL2c-dsRNA solution. (**C**) The relative expression of the *SlDCL2c* gene in tomato fruits inoculated with *SlDCL2c*-dsRNA solution for 1, 2, and 3 days. (**D**) The silencing effects of the tomato fruits inoculated with *SlDCL2c-*, *SlDCL2c-1-*, and *SlDCL2c-2*-dsRNA solution. (**E**) The expression levels of the homologous genes *SlDCL2a*, *SlDCL2b*, and *SlDCL2d* in tomato fruits inoculated with *SlDCL2c*-dsRNA solution. Fruits in the mock groups were treated with DEPC-treated water.

To investigate the action of DCL protein in 5-Aza enhanced host defense against microorganisms, the expression levels of four genes—*SlDCL2a*, *SlDCL2b*, *SlDCL2c*, and *SlDCL2d*—was quantified in tomato fruits treated with 5-Aza. The expression levels of *SlDCL2 a-d* were observed upregulated following 5-Aza treatment, showing increases of 1.25, 3.53, 7.06, and 6.36 times higher compared to those in the control group, respectively (Fig. 3A). To validate whether SIGS could trigger the silencing of target genes in postharvest fruit, tomato fruit at the red ripe stage was wounded and inoculated with *SlDCL2c*-dsRNA solution for 24 h. It was observed that the spraying of *SlDCL2c*-dsRNA significantly silenced the target genes in postharvest tomato fruits. In detail, the efficiency of gene silencing of *SlDCL2c* by *SlDCL2c*-dsRNA solution is correlated with its concentration, with the highest efficiency observed at a concentration of 200 ng/μL (Fig. 3B). Treatment with dsRNA-*SlDCL2c* at concentrations of 100, 200, and 500 ng/μL resulted in a significant reduction in the expression of the *SlDCL2c* gene, with decreases of 48%, 59%, and 51%, respectively (Fig. 3B). Conversely, treatments with 1000 ng/μL of dsRNA did not significantly suppress the gene expression. Therefore, the silencing effect of *SlDCL2c*-dsRNA in fruit exhibited a pattern of initial increase followed by a decrease as the concentration of dsRNA increased.

Next, we investigated the silencing effect of dsRNA-*SlDCL2c* on tomato fruit over time by quantifying the *SlDCL2c* gene rexpression (Fig. 3C). Following treatment of the fruit with the dsRNA solution at 200 ng/μL for 1 and 2 days, the expression of the *SlDCL2c* gene in tomato decreased significantly by 59% and 57%, respectively. However, after 3 d of treatment, the expression of the *SlDCL2c* gene in tomato fruit decreased by only 14%, showing no significant change compared to the control. The gene silencing effect on tomato fruit was most pronounced on the first and second days post-treatment, with a weakening effect observed on the third day, indicating the gene silencing effect of dsRNA-*SlDCL2c* in tomato fruit exhibited a diminishing trend over time (Fig. 3C).

To characterize the specificity of gene silencing by dsRNAs in tomato fruit, we created the other two dsRNA-*SlDCL2c* products at the best target region of CDS of *SlDCL2c* gene (NM_001329413, Table S2, see online supplementary material). Among these dsRNA-*SlDCL2c* products, the *SlDCL2c, SlDCL2c-1,* and *SlDCL2c-2* mutants achieved silencing effects of 38%, 22%, and 47%, respectively (Fig. 3D). Moreover, the expression levels of the homologous genes *SlDCL2a, SlDCL2b,* and *SlDCL2d* were also monitored to determine if there is any cross-reactivity with targets showing sequence similarity (Fig. 3E). The expression of genes *SlDCL2a, SlDCL2b,* and *SlDCL2d* was not downregulated in the dsRNA-treated fruits, indicating that there were no off-target effects observed in these studied homologous genes.

### Dicer-like protein DCL2c regulates host defense in tomato fruit against gray mold

**Figure 4:**
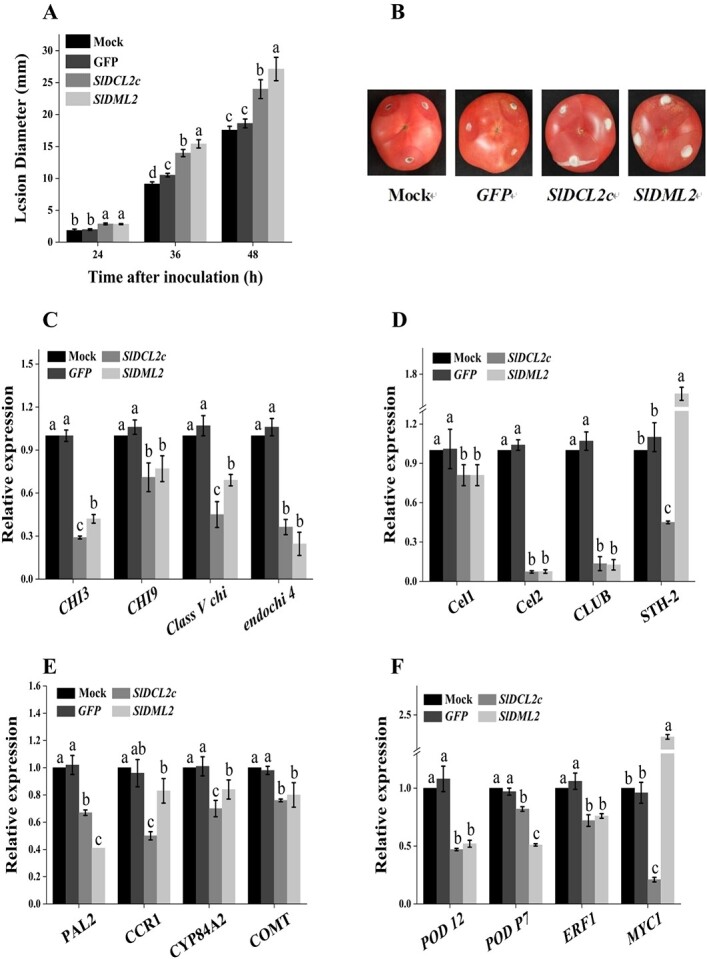
Dicer-like protein *DCL2c* regulates host defense in tomato fruit against gray mold. (**A**) The lesion diameters and (**B**) disease incidence of tomato fruits were assessed in the mock, *GFP*, *SlDCL2c*, and *SlDML2* silencing fruits after inoculation with *Botrytis cinerea*. The relative expression levels of (**C**) chitinase-related regulatory genes (*CHI3, CHI9, class V chitinase, endochitinase 4*), (**D**) beta-glucosidase-related regulatory genes (*Cel1, Cel2, GLUB*), (**E**) phenylalanine ammonia-lyase-related regulatory gene (*PAL2*), *STH-2, CCR1, CYP84A2, COMT* in the phenylpropanoid biosynthesis pathway, (**F**) peroxidase-related regulatory genes (*peroxidase 12, peroxidase P7*), transcription factors *ERF1* and *MYC1* were analysed in the mock, *GFP, SlDCL2c,* and *SlDML2* silencing tomato fruits. The mock group was treated with DEPC-treated water, and the *GFP* group was treated with a *GFP*-dsRNA solution serving as the negative control; *SlDML2* was designated as the positive control.

To investigate whether *DCL2c* contributed to 5-Aza enhanced disease resistance of tomato fruit against gray mold, tomato fruit was wounded and inoculated with *SlDCL2c*-dsRNA solution for 24 h. After dsRNA application, tomato fruit was challenged with *B. cinerea*. Fruits treated with *DCL2c*-dsRNA showed increased disease symptoms and lesion size compared to water and *GFP*-dsRNA-treated controls (Fig. 4A and B). The lesion diameters in the control group were 1.84 mm, 9.12 mm, and 17.55 mm at 24 h, 36 h, and 48 h post-inoculation, respectively. In contrast, the lesion diameters of tomato fruits treated with *SlDCL2c*-dsRNA were 2.86 mm, 13.96 mm, and 23.97 mm, respectively, representing increases of 0.55, 0.53, and 0.37 times, respectively (Fig. 4A). DEMETER-like (DML) DNA demethylase gene *DML2*, the homologue of *Arabidopsis Repressor of Silencing 1 (AtROS1)* encoding a 5mC DNA glycosylase, plays a significant role in promoting the expression of ripening-related genes and disease resistance by actively demethylating DNA [18, 23]. Here, *SlDML2* was designated as the positive control, and the absence of *SlDML2* function notably compromised the resistance of tomato fruits to the necrotrophic fungal pathogen *B. cinerea* (Fig. 4A). Therefore, these results indicated the loss of *DCL2c* also decreased disease resistance of tomato fruit against gray mold.

To investigate the specific role of the *DCL2c* gene in the host defense of tomato fruit against microorganisms, the expression levels of disease-related protein genes were quantitatively analysed in tomato fruits treated with the *SlDCL2c*-dsRNA solution. Compared to the water and *GFP*-dsRNA-treated controls, four chitinase-related regulatory genes (*CHI3, CHI9, class V chitinase, endochitinase 4*), three beta-glucosidase-related regulatory genes (*Cel1, Cel2, GLUB*), and phenylalanine ammonia-lyase-related regulatory gene (*PAL2*), peroxidase-related regulatory genes (*peroxidase 12, peroxidase P7*), and *STH-2, CCR1, CYP84A2, COMT* in phenylpropanoid biosynthesis pathway were significantly downregulated in *SlDCL2c*-dsRNA-treated tomato fruit (Fig. 4C–F). Suppression of *SlDCL2c* through dsRNA-induced RNAi resulted in a decrease in the expression of *chitinases CHI3, CHI9, Class V chitinase,* and *endochitinase 4* by 71%, 29%, 55%, 64%, as well as glucanases *Cel1, Cel2,* and *GLUB* by 19%, 93%, and 87%, respectively (Fig. 4C and D). The expression levels of genes *PAL2*, *peroxidase POD 12, POD P7, CCR1, CYP84A2*, and *COMT* in phenylpropanoid biosynthesis pathway also decreased by 33%, 53%, 18%, 50%, 30%, and 24% in *SlDCL2c*-dsRNA-treated fruit (Fig. 4E and F). These findings indicated *DCL2c* regulates pathogenesis-related proteins and phenylpropanoid biosynthesis in tomato fruit against the challenge of gray mold.

In addition, we also focus on ethylene and jasmonic acid-mediated defense and wounding responses via detection of the expression of transcription factor *ERF1* (LOC606712) and *MYC*1. Suppression of *SlDCL2c* via dsRNA-RNAi resulted in a decrease in the expression of *ERF1* and *MYC*1 by 28% and 79%, respectively (Fig. 4F). In contrast, the absence of *SlDML2* function only inhibits the accumulation of *MYC1* mRNA, indicating there are overlapping disease resistance regulatory functions of *SlDCL2c* and *SlDML2*, but there are also differences present.

**Figure 5:**
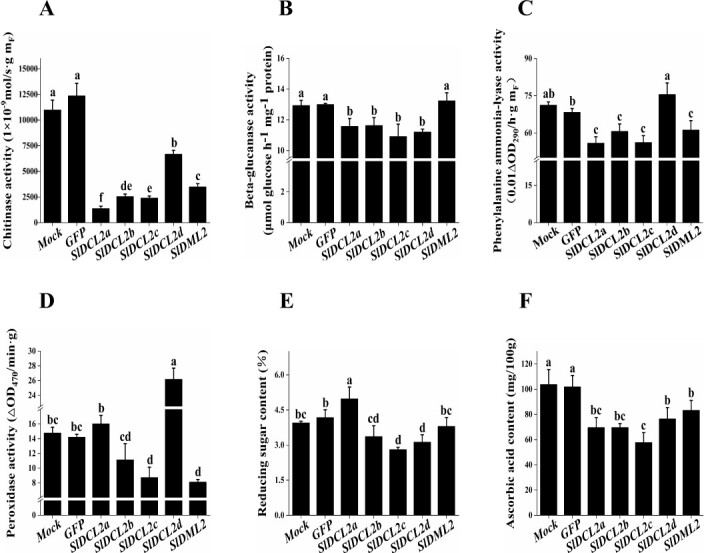
The influence of *DCL2* family genes on resistance-related enzyme activity and fruit quality in tomatoes. Chitinase (**A**), beta-glucanase (**B**), phenylalanine ammonia-lyase (**C**), and peroxidase (**D**) activities, as well as the levels of ascorbic acid and reducing sugars, were measured in the tomato fruits of the *mock, GFP, SlDCL2a, SlDCL2b, SlDCL2c, SlDCL2d*, and *SlDML2* silencing tomato fruits. The mock group was treated with DEPC-treated water, and the GFP group was treated with a GFP-dsRNA solution as the negative control; *SlDML2* served as the positive control.

We next assessed the resistance-related enzyme activity and quality indicators, such as CHI and soluble solids content, in the dsRNA-RNAi tomato fruits of *SlDCL2a, SlDCL2b, SlDCL2c*, and *SlDCL2d*. The activities of CHI and GLU in the tomato fruits applied with *SlDCL2a*-*, SlDCL2b*-*, SlDCL2c*-*,* and *SlDCL2d*-dsRNAs were significant reduced, with decreases of 87.36%, 76.75%, 78.08%, 73.45%, and 10.51%, 10.12%, 15.61%, 13.29%, respectively (Fig. 5A and B). The CHI activity in the tomato fruits of the *SlDCL2a*-dsRNA-treated fruit decreased significantly compared to the other three treatments. The PAL enzyme activity in the tomato fruits of the *SlDCL2a*-*, SlDCL2b*-*,* and *SlDCL2c*-dsRNA-treated fruit exhibited noteworthy decreases, with reductions of 21.49%, 14.80%, and 21.09%, respectively (Fig. 5C). Notably, the POD enzyme activity in the tomato fruits of the *SlDCL2c*-dsRNA-treated fruit decreased significantly by 40.83%, while the other treatment groups did not show a significant difference compared to the control group (Fig. 5D). Taken together, these results confirmed that *DCL2c* regulates pathogenesis-related proteins, phenylpropanoid biosynthesis, defense and wounding responses in tomato fruit against the challenge of gray mold.

Furthermore, a comparison with the control fruits revealed significant reductions in the ascorbic acid content in the tomato fruits of the *SlDCL2a*-dsRNA fruit, reductions in reducing sugar content and ascorbic acid content in the tomato fruits of the *SlDCL2b*-, *SlDCL2c*-, and *SlDCL2d*-dsRNA fruit, (Fig. 5E and F), indicating that the regulation of the *DCL2* gene family was also involved in the development of fruit quality.

## Discussion

Epigenetic mechanisms, such as DNA methylation and histone modifications, are intricately linked to chromatin states, and they play a crucial role in regulating gene expression linked to defense against biotic stress. Less is known about the defense response of fruit underlying epigenetic regulation against postharvest pathogenic fungi. Here, we demonstrated that DNA demethylation reduces the susceptibility of tomato fruit to gray mold by up-regulating *DCL2c*-mediated inducible defense responses. Several pieces of evidence support this claim. Firstly, coating tomato fruits with the DNA methyltransferase inhibitor 5-Aza significantly increased the activities of inducible defense enzymes CHI, GLU, and PAL, leading to a decreased susceptibility to the pathogenic fungus *B. cinerea*. Secondly, the expression levels of *SlDCL2a*–*d* were upregulated following the 5-Aza treatment. Suprression of *SlDCL2c* through SIGS resulted in the down-regulation of genes encoding PR proteins, phenylpropanoid biosynthesis and gray mold resistance in tomato fruit. Furthermore, the regulation of the *DCL2* gene family was also implicated in the development of fruit quality. This study presents evidence that DNA demethylation contributes to the immune response of tomato fruit against necrotrophic pathogens, potentially leading to prime the transcriptional activation of defense genes linked to *DCL2c*.

The precise balance between fruit ripening and defense is crucial for maintaining proper cellular function and adapting to changing conditions [24]. This balance is regulated by an integrated set of genetic and epigenetic factors, and hormonal, which coordinate the developmental process. The epigenome functions as a developmental switch, limiting the activities of ripening regulators before seed maturation in the model fruits of tomato, orange, and strawberry [15, 25, 26]. However, limited information has been collected regarding the defense response of fruit and its underlying epigenetic regulation against postharvest decay. In this study, we observed coating tomato fruits with the DNA methyltransferase inhibitor 5-Aza significantly increased the activities of inducible defense enzymes CHI, GLU, and PAL, leading to an increased disease resistance of tomato fruit to the pathogenic fungus *B. cinerea*. Consistently, Zhou *et al.* reported that the loss of *SlDML2* function reduced the resistance of tomato fruits to *B. cinerea* infection, uncovering an essential gene regulatory mechanism controlled by DNA methylation upon the necrotrophic fungal pathogen challenge [23]. Gene *SlβCA3*, which encodes a β-carbonic anhydrase, as well as *SlFAD3*, encoding a ω-3 fatty acid desaturase, are transcriptionally activated through *SlDML2*-mediated DNA demethylation, and were positively regulating tomato resistance to *B. cinerea*, likely functioning within the same genetic pathway as *SlDML*2 [23]. Taking into account the previous study and our findings, DNA demethylation positively regulates the defense against pathogen *B. cinerea* in tomato fruit.

Fruit development is an elegant evolutionary strategy unique to angiosperms, serving the dual purpose of protecting developing seeds and facilitating their dispersal [27]. When fruits ripen, they typically have higher sugar content and nutrient levels, and their resistance to herbivores decreases, enhancing their attractiveness to fruit-eating animals. Simultaneously, this ripening process creates a favorable environment for fungal growth, which is considered a major factor triggering decay in ripening fruit [16]. However, a distinctive observation in this phenomenon under the present investigation is the documented increase in defense responses against necrotrophic pathogens, including PAL, CHI, 4-coumarate CoA ligase, peroxidase, GLU, and other factors during fruit ripening [18]. Mutant *jin1* and *jar1–1* plants, which are impaired in the MYC2 branch of the jasmonic acid pathway involved in responses to herbivores, exhibited significantly increased expression of both ORA59 and PDF1.2, which are important in defense against necrotrophic pathogens [28]. Plant defenses against insect herbivores and necrotrophic pathogens are regulated differently by distinct branches of the jasmonic acid signaling pathway. Mutations or downregulation of DNA methylation pathways, as observed in *Arabidopsis* (*met1–3* and the triple *drm1–2 drm2–2 cmt3–11* mutants) and rice, results in elevated levels of pathogen resistance and an increased expression of defense-related genes [29–31]. Hence, the strategy of safeguarding tomato fruit through DNA demethylation represents a neglected innovative management technique for reducing postharvest fungal diseases.

DCL proteins are key components in small RNA biogenesis, including miRNAs and siRNAs, which were involved in plant resistance, e.g., miR393, miR319c, miR482b, miR1127-3p, siR109944, and lncRNA4504 [32]. In tomato, the expression levels of *SlDCLs* varied significantly among leaves, flowers, and fruits at different stages of growth [33]. All seven *SlDCLs* (*DCL1, DCL2 a-d, DCL3,* and *DCL4*) were found to be expressed in all organs, with flowers (*SlDCL1*) and mature green fruits (*SlDCL2c*), and peaking at the breaker stage (*SlDCL2a*) showing markedly higher expression levels compared to other parts. The expression of *SlDCL3* and *SlDCL4* was notably low in the immature stage, and their expression patterns varied within fruits [33]. Moreover, tomatoes encode four functional *DCL* family genes, each potentially playing distinct roles in regulating plant growth and development across different plant species, with *DCL2* being relatively understudied [34, 35]. Here, a significant upregulation of *SlDCL2c* was observed following 5-Aza treatment. Suppression of *SlDCL2c* through SIGS resulted in a decrease in disease resistance in tomato fruit against pathogenic fungi *B. cinerea*, indicating *DCL2* not only plays a significant role in fruit development and ripening, but is also involved in resistance against fungal diseases. Similarly, the knockout of *SlyDCL2b* resulted in increased TMV accumulation in the leaf primordium. In *slydcl2b* mutants, 22-nt virus-derived small interfering RNAs accumulated less abundantly compared to wild-type plants [33]. In tomato fruit, suppression of *DCL2c* also inhibited the PR proteins, phenylpropanoid biosynthesis, defense and wounding responses. Four chitinase-related regulatory genes (*CHI3, CHI9, class V chitinase, endochitinase 4*), three beta-glucosidase-related regulatory genes (*Cel1, Cel2, GLUB*), and *PAL2*, *peroxidase 12, peroxidase P7*, *STH-2, CCR1, CYP84A2,* and *COMT* in phenylpropanoid biosynthesis pathway were significantly downregulated in *SlDCL2c*-dsRNA-treated tomato fruit. Consistent with our findings is the loss-of-function *dcl2b* mutant was challenged with tomato mosaic virus, the genes related to lipid metabolism, secondary metabolic and oxidation–reduction were suppressed [32]. Overall, *DCL2c*, which has close ties with fruit ripening, regulates the responses in tomato fruit against the pathogenic fungus *B. cinerea* challenge.

In fresh fruit and vegetables, RNA interference (RNAi) technology has been extensively studied for its potential to enhance nutrient content, reduce harmful substances, improve resistance to browning and softening, with a primary focus on transgenic plant production. By constructing plants that can stably produce dsRNA in potato chloroplasts, Zhang *et al.* significantly improved the insect resistance of potato plants [36]. In February 2015, the United States Department of Agriculture (USDA) approved the world’s first *PPO* dsRNA-based transgenic non-browning Arctic® apple variety developed by Okanagan Specialty Fruits (OSF) [37]. In the same year, Simplot, a supplier of McDonald’s, used RNAi to inhibit the expression of *ASN1* and *PPO5* genes in potatoes to produce non-browning potatoes, with reduced dark spots and reduced acrylamide (a carcinogen) content, and genetic modification (GM) safety evaluations and commercial applications have been approved in five countries, including the United States, Canada, and Australia [38]. Besides, the introduction of dsRNA of 1-Aminocyclopropane-1- carboxylate (ACC) oxidase gene in tomato delayed ethylene production rate in ripened fruits, ensuring a prolonged shelf life of tomato [39]. However, the acceptance of RNAi-mediated transgenic plants has faced challenges due to concerns regarding their potential impacts on human health and the environment [40]. Recently, the utilization of exogenously applied dsRNAs, short interfering RNAs (siRNAs), and hairpin RNAs (hpRNAs) has emerged as an alternative approach that may be perceived as more environmentally friendly, sustainable, and socially acceptable than genetic modification. The application of dsRNA-ERG reduced *B. cinerea* germination and growth in *in vitro* conditions and various fruits, such as strawberries, mango, onion scale, cherry, rose petals, bell pepper, and grape, resulting in a reduction of gray mold decay [41]. Application of dsRNAs targeting *PPO* and *PALX* genes led to a reduction in their expression levels in potatoes, resulting in decreased activities of polyphenol oxidase and phenylalanine ammonia-lyase enzymes, which in turn led to reduced fresh-cut browning [42]. Here, tomato fruit at the red ripe stage was wounded and inoculated with *SlDCL2*-dsRNA solutions to trigger the silencing of target genes in postharvest tomato fruit, and it was observed that the spraying of *SlDCL2*-dsRNAs significantly silenced the target genes in postharvest tomato fruits. Therefore, utilizing dsRNA preparations to silence host genes in fruit can enhance quality attributes, as well as improve resistance to fungal and viral infections. Notably, when dsRNA preparations are applied to fresh fruit and vegetables, it is important to consider the efficiency and stability of dsRNA silencing.

## Conclusion

The strategy of protecting tomato fruit through DNA demethylation involves the activation of PR proteins, phenylpropanoid biosynthesis, and hormone signaling. This strategy constitutes an innovative management approach to controlling postharvest fungal diseases by modulating multiple genes responsible for infection by necrotrophic pathogens, as opposed to relying on existing methods that induce disease resistance activation. The key component in the biogenesis of 22-nt small RNAs, *DCL2c*, regulates the responses of tomato fruit to the challenge posed by the pathogenic fungus *B. cinerea*. Silencing of *SlDCL2c* through SIGS resulted in the down-regulation of genes encoding PR proteins, phenylpropanoid biosynthesis and gray mold resistance in tomato fruit. Furthermore, the regulation of the *DCL2* gene family was also implicated in the development of fruit quality. Taken together, this study presents evidence that chemical induction of DNA demethylation by 5-Aza enhances tomato fruit defense against gray mold through dicer-like protein-encoding gene *DCL2c*.

## Materials and methods

### Fruit material

*Solanum lycopersicum* (tomato) fruits of the cultivar ‘Jingcai 8’ were hand harvested at the red ripe stage from Beijing Mishui Agricultural Production and Marketing Cooperatives in Beijing City, China. Before transportation to the laboratory, all fruits were inspected for injuries and infections. To ensure sterilization, the fruits underwent surface treatment with a 1% (v/v) sodium hypochlorite solution for 2 minutes, followed by rinsing with tap water and air-drying at room temperature.

The *S. lycopersicum* fruits were coated with 5-Azacytidine (A2385, Sigma-Aldrich/Merck, China) at varying concentrations (0, 25, 50, 100 mmol/L) for two applications, with a two-day interval between each. Following storage at 90–95% relative humidity and 28°C for 5 d, samples (1–3 g) were collected and frozen at −80°C for later use. Changes in color and firmness of the fruits were monitored daily (day 0–5) after the two coatings with 50 mmol/L of 5-Aza. To trigger gene silencing using dsRNA, fruits were wounded using a sterile borer to create three wounds, each 2 mm deep and 5 mm in diameter on the surface. Following this, five microliters of the dsRNA solution were applied to each wound, while the control group received sterile distilled water. After being stored at 90–95% relative humidity and 28°C for 24 hours, samples weighing 1–3 g each were collected and frozen at −80°C for subsequent experiments. The pathogen *Botrytis cinerea* was inoculated into the 5-Aza- or dsRNA-treated fruits to evaluate their resistance to mold infection.

### Microbial culture and infection assay

The *B. cinerea* culture and infection assay were conducted following the method outlined by Lu *et al.* with minor adjustments [43]. *B. cinerea* was cultured for 7 days on potato dextrose agar (PDA) at a concentration of 200 g L^−1^, maintained at 28°C in the dark. The spores were collected, washed with sterile water, prepared into a spore suspension, and then quantified microscopically, adjusting the concentration to 1 × 10^5^ spores mL^−1^. *S. lycopersicum* fruits were wounded using a sterile borer to create three wounds, each 2 mm deep and 5 mm in diameter, on the surface. Thirty microliters of the *B. cinerea* suspension were then added to each wound, while sterile distilled water was used for control inoculations. The percentage of infected wounds and the lesion diameter were recorded at different time interval post-storage under conditions of 90–95% relative humidity and 28°C. The antifungal activity of 5-Aza was assessed against the fungal pathogen using the agar diffusion plate method. A volume of 100 μL of *B. cinerea* suspension (with a concentration of approximately 1 × 10^4^ spores mL^−1^) was evenly spread on the surface of the PDA solid culture plate. Subsequently, a filter paper saturated with 5-Aza was placed on the PDA solid medium, and the diameter of the inhibition zone was measured after a 24-hour incubation period at 28°C.

### Design and construction of dsRNA

The construction and production of dsRNA were conducted following the method outlined by Chen *et al.* with minor adjustments [42]. Five dsRNA sequences were designed to target *SlDCL2a* (NM_001329410.1), *SlDCL2b* (NM_001329412.1), *SlDCL2c* (NM_001329413.1), *SlDCL2d* (NM_001329414.1), and *SlDML2* (XM_004249411.4) genes, with *SlDML2* utilized as a positive control. The dsRNAs were designed to target specific regions of the corresponding coding DNA sequences using the SGN VIGS Tool online platform (https://vigs.solgenomics.net/). Parameters for selection included an n-mer of 21 bp, fragment length of 500 bp, zero mismatches, and *S. lycopersicum* ITVG v2.40 as the database. The optimal target regions and scores (−∞-100) for *SlDCL2a, SlDCL2b, SlDCL2c, SlDCL2d*, and *SlDML2* were identified as 2124–2623 (23.20), 1448–1947 (76.60), 804–1303 (35.85), 2106–2605 (53.50), and 1–500 (100.00). Only an additional off-target, *Ribonuclease 3-like protein 3*, was detected. To ensure specificity of the target template, BLAST technology was employed, restricting the organism to *S. lycopersicum*. Various *SlDCL2c* dsRNAs were introduced at distinct loci to evaluate their gene silencing efficacy on tomato fruits. The expression levels of the corresponding genes *SlDCL2a, SlDCL2b,* and *SlDCL2d* were assessed in the silenced tomato fruits of *SlDCL2c* to validate any off-target effects of RNAi. Primer pairs used for dsRNA synthesis consisted of T7 polymerase recognition sequence and transcriptional promoters, and gene-specific primers. The gene-specific primers were designed utilizing the NCBI BLAST program and were synthesized by GENEWIZ (Tianjin, China), with specific information available in Table S2 (see online supplementary material).

Total RNA was extracted from approximately 0.1 g of tomato samples using RNAiso Plus (Item No. 9109, TaKaRa, Dalian, China) following the manufacturer’s instructions. Reverse transcription was conducted using the PrimeScript® RT reagent kit with gDNA eraser (Item No. RR047A, Dalian, TaKaRa, China) to convert RNA into complementary DNA (cDNA). The RT-PCR products were purified through gel electrophoresis using the MiniBEST Agarose Gel DNA Extraction Kit Ver. 4.0 (Item No. 9762, Dalian, TaKaRa, China) following the manufacturer’s instructions. The purified PCR product was used as the template for dsRNA synthesis, conducted at 42°C for 2–4 h following the manufacturer’s protocol with the In vitro Transcription T7 Kit (Item No. 6140, Dalian, TaKaRa, China). For the negative control, the GFP fragment from a pTRV2-GFP plasmid (HonorGene, Code No. HG-VRH1165) was amplified. The dsRNA was subsequently purified using NucleoSpin RNA Clean-up (Item No. 740948.10, Dalian, TaKaRa, China) according to the instructions provided. Quality assessment of the dsRNA was performed using NanoDrop and agarose gel electrophoresis. The dsRNA was stored at −80°C until needed.

### Gene expression assays

Gene expression assays were conducted using qRT-PCR analysis following the method by Zhang *et al.* with slight modifications [44]. The assays were performed with the TB Green Premix Ex Taq II (TaKaRa) and CFX384 system (Bio-Rad, Hercules, CA, USA). The qRT-PCR reaction mixture included 10 μL of TB Green Premix Ex Taq II, 0.8 μL of gene-specific primer pairs, 0.4 μL of ROX Reference Dye or Dye II, 2 μL of 10× diluted cDNA sample, and 6 μL of nuclease-free water for a total volume of 20 μL. The PCR program comprised an initial step at 95°C for 30 s, followed by 40 cycles of 94°C for 5 s, 50°C for 30 s, and 72°C for 60 s. Melting curves were generated at the end of the cycling by gradually increasing the temperature from 55°C to 95°C in 0.5°C increments every 5 s to assess primer specificity. Specific primers are detailed in Table S3 (see online supplementary material).

### Enzyme activities assay

Chitinase activity was assessed following the method described by Hong *et al.* with slight modifications [45]. The assay mixture comprised 500 μL of 10 g/L colloidal chitin, 500 μL of sodium acetate buffer (pH 5.2) and enzyme extract, and 40 μL of 30 g/L snailase, and was then incubated at 37°C for 2 h. After the addition of 80 μL of potassium tetraborate, the reaction mixture was heated in boiling water for 3 minutes. Subsequently, the mixture was incubated with dimethylamine borane at 37°C for 20 minutes. The absorbance at 540 nm was recorded to ascertain chitinase activity by measuring the levels of reducing sugar, specifically N-acetyl-d-glucosamine (NAG). The chitinase activity unit (U) is defined as the quantity of enzyme that releases 1 μmol of NAG per hour.

*Beta*-glucanase activity was assessed following the method outlined by Gholamnezhad with slight modifications [46]. The reaction mixture consisted of 500 μL of 0.04% laminarin dissolved in a 0.1 mmol L^−1^ sodium acetate buffer with a pH of 5.2, along with 500 μL of fruit extract, and was incubated at 50°C. After 10 minutes, the reaction was halted by adding 1 mL of dinitro-salicylic acid reagent and heating for 5 minutes, followed by the dilution of the reaction mixture with 5 mL of distilled water. The absorbance of the resulting color was recorded at 540 nm. A standard curve correlating the absorbance at 540 nm with glucose concentration was utilized to measure enzyme activity. The *Beta*-glucanase activity unit (U) is defined as the amount of enzyme necessary to release 1 μmol of glucose equivalent per hour under the specified assay conditions.

The activity of phenylalanine ammonia-lyase was measured following the method outlined by Wang *et al.* with minor modifications [47]. A mixture containing 1 mL of the extraction buffer, 0.5 mL of 10 mM L-phenylalanine, 0.4 mL of double-distilled water, and 0.1 mL of enzyme extract was incubated at 37°C for 1 hour. The reaction was halted by adding 0.5 mL of 6 M HCl, and the concentration was determined by measuring the absorbance at 290 nm. The unit (U) of phenylalanine ammonia-lyase activity is defined as the production of 1 μmol of cinnamic acid per minute.

The pectinesterase activity was assessed following the procedure outlined by Terefe *et al.*, with slight modifications [48]. A 500 μL portion of crude enzyme extract was combined with a 1% apple pectin solution, pre-equilibrated to 37°C. The pH of the reaction mixture, maintained at 7.5, was controlled by titrating with 0.01 N NaOH solution during the pectin hydrolysis at 37°C. The consumption of NaOH was monitored over a 30-minute reaction period. The unit (U) of pectinesterase activity is defined as the amount of enzyme required to release 1 μmol of carboxyl group per hour under the assay conditions.

The peroxidase activity was measured by mixing 500 μL of the supernatant with 3 mL of a 100 mmol L^−1^ sodium phosphate buffer at pH 5.5 containing 0.56% (v/v) of guaiacol [49]. The reaction was initiated by adding 0.2 mL of 0.5 mol L^−1^ hydrogen peroxide. After an incubation at 25°C for 15 seconds, the absorbance at 470 nm was recorded. The peroxidase activity unit (U) is defined as an increase of 1 in the absorbance change value per minute per gram of sample.

### Assessments of fruit quality

The variations in color (L*, a*, b* values) of tomato fruits during storage were evaluated using a portable handheld colorimeter (WR-18, Shenzhen WaveGD Photoelectric Technology Co., Shenzhen, China) following the procedure outlined by Qiao *et al.* [50]. Fruit firmness was measured with a hardness tester (GY-4, Aidebao Instruments Co., Ltd, Zhejiang, China). Soluble solids, reducing sugars, and ascorbic acid levels were measured in accordance with the procedure outlined by Anar *et al.*, with minor modifications [51]. Total soluble solids in all tomato fruits were determined using a digital refractometer (AP-55, Aipu Instrument Co., Ltd, Shenzhen, China). Reducing sugars were quantified using the dinitrosalicylic acid method. To determine the ascorbic acid content in mg/100 g, a specific volume of filtered juice was diluted with 3% metaphosphoric acid and titrated against 2,6-dichlorophenol indophenol until a stable light pink color was attained.

### Data analysis

The experimental data were analysed using IBM SPSS (IBM SPSS Statistics 27 software, SPSS Inc., Chicago, IL, USA). An independent samples *t*-test was employed to analyse differences between two sets of data, while one-way ANOVA and Duncan's test (*P* < 0.05) were used for comparisons involving three or more sets of data. The experimental results are presented as the mean ± standard error of three repeated samples. Graphs were created using Origin 2021 (Origin 2021, OriginLab Corporation, Northampton, MA, USA).

## Supplementary Material

Web_Material_uhae164

## Data Availability

The data underlying this article are available in the article and in its online supplementary material. More data underlying this article will be shared on reasonable request to the corresponding author.
