# Supplementary material for: Chemical induction of DNA demethylation by 5-Azacytidine enhances tomato fruit defense against gray mold through dicer-like protein DCL2c
Source: Hortic Res. 2024 Jun 19;11(8):uhae164. doi: 10.1093/hr/uhae164 (PMC11298621; doi:10.1093/hr/uhae164)
Supplement: Web_Material_uhae164 [file web_material_uhae164.zip › Supplementary Table S1-S3.doc]

**SUPPORTING INFORMATION**

**Chemical Induction of DNA Demethylation by 5-Azacytidine Enhances Tomato Fruit Defense against Gray Mold through Dicer-like Protein *DCL2c***

Xiaorong Chang, Liyao Liu, Ziwei Liu, Liping Qiao, Ruixi Shi, Laifeng Lu*

*Tianjin Key Laboratory of Food Quality and Health, State Key Laboratory of Food Nutrition and Safety, College of Food Engineering and Biotechnology, Tianjin University of Science and Technology, Tianjin 300457, P.R. China*

** Corresponding author. Tianjin University Science and Technology, No. 29,13th Street, Tianjin 300457, P. R. China. Tel: +86-022-60912453; Fax: +86-022-60912453; E-mail: Frank@tust.edu.cn*

**Table S1 The impact of chemical induction of DNA demethylation by 5-azacytidine on the quality of tomato fruits.**

|  | **Dose of 5-azacytidine (m moL/L)** | | | |
| --- | --- | --- | --- | --- |
|  | **0** | **25** | **50** | **100** |
| *L** value | 48.55±0.47 ^a^ | 47.22±0.94 ^a^ | 47.78±0.14 ^a^ | 47.5±1.5 ^a^ |
| *a** value | 6.46±0.91 ^b^ | 11.71±1.12 ^a^ | 10.36±1.56 ^a^ | 10.99±0.40 ^a^ |
| *b** value | 18.94±0.84 ^a^ | 18.43±0.1.06 ^a^ | 16.91±0.28 ^ab^ | 15.96±1.94 ^b^ |
| Firmness (N) | 31.45±1.27 ^a^ | 28.52±1.23 ^b^ | 24.32±1.12 ^c^ | 22.71±1.00 ^c^ |
| Reducing sugar (%) | 1.70±0.06 ^c^ | 1.85±0.04 ^b^ | 1.97±0.10 ^ab^ | 2.04±0.04 ^a^ |
| Ascorbic acid (mg/100g) | 148.11±10.95 ^c^ | 301.00±21.50 ^b^ | 315.33±14.33 ^b^ | 394.17±18.96 ^a^ |
| Total Soluble Solids (%) | 4.43±0.09 ^b^ | 4.93±0.15 ^a^ | 4.74±0.19 ^a^ | 4.43±0.12 ^b^ |

* Different lowercase letters indicate significant differences according to Duncan’s multiple comparison test.

**Table S2** The primer sequences used for double-stranded RNA synthesis.

| **Gene_id** | **Gene_name** | **Gene_description** | **Forward primer** | **Reverse primer** |
| --- | --- | --- | --- | --- |
| NM_001329410.1 | *SlDCL2a* | endoribonuclease Dicer 2a | GATCACTAATACGACTCACTATAGGGAGGGGACGCTTACAAGTTCAA | GATCACTAATACGACTCACTATAGGGTCACCCCAGTTTTGGTGCTA |
| NM_001329412.1 | *SlDCL2b* | endoribonuclease Dicer 2b | GATCACTAATACGACTCACTATAGGGATGGCCAGTGGGGAGATGAT | GATCACTAATACGACTCACTATAGGGGATTGTCAGTCAACGCACCC |
| NM_001329413.1 | *SlDCL2c* | endoribonuclease Dicer 2c | GATCACTAATACGACTCACTATAGGGAGTGAGATGGGTGTGTGGCT | GATCACTAATACGACTCACTATAGGGACAGAAACATGCCCTGCTGTA |
|  | *SlDCL2c-1* |  | GATCACTAATACGACTCACTATAGGGGGCAGCTGAGTTCTTGTCCTT | GATCACTAATACGACTCACTATAGGGGCACAGAAACATGCCCTGCT |
|  | *SlDCL2c-2* |  | GATCACTAATACGACTCACTATAGGGATCGAAACTTCACATGGCTTTCT | GATCACTAATACGACTCACTATAGGGTTCGTTCCGGAAGCAACTC |
| NM_001329414.1 | *SlDCL2d* | endoribonuclease Dicer 2d | GATCACTAATACGACTCACTATAGGGTTCGGATGGGAGGCTGAAAC | GATCACTAATACGACTCACTATAGGGAGCCACCTTCTCAGCCATTT |
| XM_004249411.4 | *SlDML2* | DNA demethylase 2 | GATCACTAATACGACTCACTATAGGGCAGGGAAGCCAAGTTTTGCC | GATCACTAATACGACTCACTATAGGGGCCAACAGATCCCCGAAAGA |

* The primer pairs used for double-stranded RNA synthesis included a T7 polymerase recognition sequence, T7 transcriptional promoters, and gene-specific primers. The T7 polymerase recognition sequence is underlined.

**Table S3 The primer sequences of defense-related genes in tomato fruit utilized in qRT-PCR analyses.**

| **Gene_id** | **Gene_name** | **Gene_description** | **Forward primer** | **Reverse primer** |
| --- | --- | --- | --- | --- |
| NM_001329410.1 | *SlDCL2a* | endoribonuclease Dicer 2a | ATGCTATCAGCGCTTGGAAT | TTGCACCACCAAGGGATTCA |
| NM_001329412.1 | *SlDCL2b* | endoribonuclease Dicer 2b | GCGCGAGCAAATGATGTTGT | CAACCCCCATTTCTCCCCAA |
| NM_001329413.1 | *SlDCL2c* | endoribonuclease Dicer 2c | CCAACCGCTACGATTCTGGA | ACGTTAGGAGCACTTCGTCG |
| NM_001329414.1 | *SlDCL2d* | endoribonuclease Dicer 2d | CGGATGGGAGGCTGAAACTA | TTGCTAGCCACCTTCTCAGC |
| [NM_001320601.1](https://www.ncbi.nlm.nih.gov/nuccore/NM_001320601.1) | *PAL2* | phenylalanine ammonia-lyase 2 | GGGGAGACGTTGACAGTAGC | CCGCCTCTGATAGTTGCACA |
| [NM_001247683.1](https://www.ncbi.nlm.nih.gov/nuccore/NM_001247683.1) | *CCR1* | cinnamoyl-CoA reductase | CATGGACCCAAGCAGAGACC | GCAATGGTCCAAGCACCAAC |
| [XM_004232410.4](https://www.ncbi.nlm.nih.gov/nuccore/XM_004232410.4) | *CYP84A2* | cytochrome P450 84A2 | TTTCGATGTCCCGCCGTAAA | CAAGTTTGGCAAGACCACGG |
| [XM_004234980.3](https://www.ncbi.nlm.nih.gov/entrez/viewer.fcgi?db=nucleotide&id=1104614274) | *LOC101251452* | caffeic acid 3-O-methyltransferase | GCGAGTGCCTCTGTACTACC | AGCAGCAAGAAGCCTAAGCA |
| [NM_001347482.1](https://www.ncbi.nlm.nih.gov/entrez/viewer.fcgi?db=nucleotide&id=1108847088) | *LOC101253377* | peroxidase 12 | TCTGTTGTCTTGACGGGTGG | CAGAGGGTGCGACTAGGTTG |
| [XM_004240835.4](https://www.ncbi.nlm.nih.gov/entrez/viewer.fcgi?db=nucleotide&id=1444424147) | *LOC101261825* | peroxidase P7 | CACTAGCGAGAACCAGGCAA | AGTGATTGTCGAAGCGCGTA |
| [NM_001323319.1](https://www.ncbi.nlm.nih.gov/entrez/viewer.fcgi?db=nucleotide&id=1020158934) | *LOC101246666* | pathogenesis-related protein STH-2 | GCTGAGGGAGATGGAAGTATCA | GACTTGCAAACACATCCTCCAT |
| [NM_001247933.2](https://www.ncbi.nlm.nih.gov/nuccore/NM_001247933.2) | *Cel1* | endo-1,4-beta-glucanase | AATGCAATTCGTTGGGGCAC | GCCACCTCAGATCCTGGAGA |
| [NM_001247938.1](https://www.ncbi.nlm.nih.gov/entrez/viewer.fcgi?db=nucleotide&id=350538696) | *Cel2* | endo-1,4-beta-glucanase precursor | CCTGCCCTCCTCAAACACAT | ATGGCACAGAGCTACCCCTA |
| [NM_001247876.2](https://www.ncbi.nlm.nih.gov/entrez/viewer.fcgi?db=nucleotide&id=917953721) | *GluB* | glucan endo-1,3-beta-glucosidase B | ATCAGCCCTGTTACTGGCAC | GGTGCACGTGTATCCCTCAA |
| [NM_001247912.2](https://www.ncbi.nlm.nih.gov/entrez/viewer.fcgi?db=nucleotide&id=906847311) | *LOC606712* | ethylene-responsive transcription factor 1 | ATGAGGGGTCCTTGGTCTCT | ACAGCAGCTGGAGATAATCCAT |
| [XM_004248589.3](https://www.ncbi.nlm.nih.gov/entrez/viewer.fcgi?db=nucleotide&id=1444437175) | *LOC101267358* | endochitinase 4 | TGGTAGACAGGCAGGAGGTG | TGTCTCCACCAGAAGGACCA |
| [NM_001247475.2](https://www.ncbi.nlm.nih.gov/entrez/viewer.fcgi?db=nucleotide&id=937834170) | *CHI3* | chitinase | CAATGGACGCCATCCCCTAA | CGGACCCATCCCACATTCAA |
| [NM_001247474.2](https://www.ncbi.nlm.nih.gov/entrez/viewer.fcgi?db=nucleotide&id=937834175) | *CHI9* | chitinase | TGTGCTTTTGCTGTCTGCCT | CTAGGTCCCCTGAAGGACCG |
| [XM_004242514.4](https://www.ncbi.nlm.nih.gov/entrez/viewer.fcgi?db=nucleotide&id=1444426866) | *LOC101257783* | class V chitinase | TTTGTGCGTTCGCAAACCTT | CTCCCTCCCCCTATGGACAA |
| [NM_001301178.2](https://www.ncbi.nlm.nih.gov/entrez/viewer.fcgi?db=nucleotide&id=815930022) | *MYC1* | transcription factor MYC1 | TGCTTCCACTCCCAATCGTC | TACCCATCTCCCCAACCCAA |
